# Metformin Exposure in Pregnancy and Childhood Developmental Outcomes

**DOI:** 10.1001/jamanetworkopen.2026.37054

**Published:** 2026-09-30

**Authors:** Hannah Gordon, Richard J. Hiscock, Sarah Price, Alexis Shub, Alexandra Roddy Mitchell, Jessica Atkinson, Susan Walker, Anna Forsythe, Stephen Tong, Anthea Lindquist, Roxanne Hastie

**Affiliations:** 1 Perinatal Epidemiology Group, Department of Obstetrics, Gynaecology and Newborn Health, University of Melbourne, Melbourne, Victoria, Australia; 2 Mercy Perinatal, Mercy Hospital for Women, Heidelberg, Victoria, Australia; 3 The Royal Women’s Hospital, Parkville, Victoria, Australia

## Abstract

**Importance:**

Metformin is an oral hypoglycemic agent increasingly prescribed during pregnancy. In animal models, metformin influences embryonic cortical development, and it is uncertain whether this translates to clinical implications for humans.

**Objective:**

To examine whether metformin prescription in pregnancy is associated with a risk of developmental vulnerability among offspring at school entry.

**Design, Setting, and Participants:**

This cohort study used population-level pregnancy and birth data from January 1, 2009, to December 31, 2020, linked with the Australian Early Development Census (AEDC), a standardized national assessment of childhood development completed every 3 years during the first year of full-time school (age 4-6 years). The data analysis was performed between March 14, 2025, and January 30, 2026.

**Exposure:**

Metformin prescription dispensation during pregnancy.

**Main Outcome and Measures:**

The association of developmental vulnerability, defined as scoring below the 10th percentile in at least 2 of the 5 assessed domains of the AEDC (physical health and well-being, social competence, emotional maturity, school-based language and cognitive skills, and communication skills and general knowledge), was estimated using inverse probability of treatment weighting combined with regression adjustment.

**Results:**

From 871 627 singleton births in Victoria between 2009 and 2020, 177 409 children with linked developmental outcome data were identified, of whom 1095 (0.6%) were exposed to metformin antenatally (43 [3.9%] aged ≤5 years 0 months, 860 [78.5%] aged 5 years 1 month to 6 years 0 months, and 192 [17.5%] aged ≥6 years 1 month at assessment; 557 male [50.9%]). Developmental vulnerability was observed in 199 children (18.2%) exposed to metformin compared with 24 379 children (13.9%) not exposed. After adjustment for confounders, metformin was not associated with developmental vulnerability among children exposed to metformin (adjusted relative risk, 0.97 [95% CI, 0.74-1.29]) or across any of the individual developmental domains.

**Conclusions and Relevance:**

This cohort study found that metformin use in pregnancy was not associated with an altered risk of developmental vulnerability among children aged 4 to 6 years and may provide reassurance to clinicians and women considering metformin use during pregnancy.

## Key Points

**Question:**

What is the association of metformin use during pregnancy with developmental outcomes among offspring during the first year of full-time school?

**Findings:**

In this cohort study of 177 409 singleton children born in Victoria, Australia, between 2009 and 2020, no association was observed between metformin use during pregnancy and childhood developmental vulnerability (scoring ≤10th percentile on ≥2 of 5 assessed developmental domains using a national assessment tool) after accounting for confounding factors.

**Meaning:**

These findings suggest that metformin in pregnancy is not associated with developmental vulnerability in children aged 4 to 6 years and may provide reassurance for clinicians and women considering metformin use during pregnancy.

## Teaser

This cohort study examines the association of antenatal metformin exposure with child development at school entry.

## Introduction

Metformin is an oral hypoglycemic medication that is first-line treatment for type 2 diabetes.^1^ The use of metformin in pregnancy is increasing.^2^ During pregnancy, metformin crosses the placenta, with the fetus exposed to similar medication concentrations as that in the maternal blood stream,^3^ raising potential concerns about fetal harm. There are reassuring, short-term perinatal safety data,^4,5^ but long-term safety for exposed offspring remains incompletely described.^6,7^ Metformin may be associated with increased adiposity in childhood,^8^ but research in this area is conflicting^9,10^ and lacks follow-up into adulthood.

Considering neurodevelopmental implications, an animal study suggested that in utero exposure to metformin may cause impaired cortical development,^11^ but it remains unclear whether this is relevant to human offspring development. A recent systematic review of 7 studies observed no association between in utero metformin exposure and global neurodevelopmental delay in children younger than 2 years (relative risk [RR], 1.09 [95% CI, 0.54-2.17]; 3 studies; 9668 children) and aged 3 to 5 years (RR, 0.90 [95% CI, 0.56-1.45]; 2 studies; 6118 children).^7^ However, in this preschool age range, developmental delays may not yet be apparent. Furthermore, individual studies in this area have been limited by small sample sizes and heterogeneous study populations, with metformin prescribed for diverse indications.^8,12^

Larger studies focusing on outcomes for school-aged children are needed to provide evidence for women and their clinicians considering metformin use during pregnancy. Using population-level data from Victoria, Australia, we investigated whether antenatal metformin exposure during pregnancy was associated with an altered risk of developmental vulnerability on a national assessment among offspring during the first year of full-time school.

## Methods

This cohort study included all singleton births in Victoria, Australia, between January 1, 2009, and December 31, 2020, with linked pregnancy and school-aged developmental outcome data. Ethical approval was received from the Mercy Health Ethics Committee and the Australian Institute of Health and Welfare. A waiver of informed consent was approved for this study. This research adhered to the Strengthening the Reporting of Observational Studies in Epidemiology (STROBE) reporting guideline for cohort studies.

The following 5 state-level and national-level databases were linked for this study by the Centre for Victorian Data Linkage and the Australian Institute of Health and Welfare: (1) Victorian Perinatal Data Collection (VPDC), (2) National Diabetes Service Scheme (NDSS), (3) Pharmaceutical Benefits Scheme (PBS), (4) Australian Early Development Census (AEDC), and (5) Victorian Admitted Episodes Dataset. Pregnancy data were sought from the VPDC, which collects statewide data related to pregnancy, birth, and perinatal outcomes, including pregnancy and birth complications, mode of birth, gestational age at birth, birth weight, and the presence of congenital anomalies. It is mandatory for every birth occurring after 20 weeks’ gestation to be recorded in the VPDC, with the database regularly audited to ensure data accuracy and completeness.^13^ Maternal medical conditions, including preeclampsia and polycystic ovary syndrome, were identified using the VPDC (eTable 1 in Supplement 1).

Birth data were linked with diabetes diagnoses from the NDSS (gestational diabetes, type 1 diabetes, type 2 diabetes). The NDSS is an Australia-wide registry including 80% to 90% of people diagnosed with diabetes^14^ and is further enriched by diabetes diagnoses identified in the VPDC. Each record in the NDSS is validated by an authorized health professional to ensure accuracy.^15^

### Exposure

Metformin exposure was identified through dispensation of at least 1 metformin prescription during pregnancy by the PBS. The PBS is an Australian government initiative that completely or partially subsidizes the cost of more than 900 medications for Australian citizens, including metformin. Under the PBS, metformin is dispensed in boxes of between 60 and 120 tablets and prescribed for 1 to 3 times a day, with a typical prescription supplying between 1 and 6 months of medication.^16^

Metformin use in pregnancy was defined as dispensing a prescription any time from the first day of the last normal menstrual period until birth. The first day of the last normal menstrual period was calculated by subtracting gestational days at birth from the child’s date of birth. Information related to trimester of exposure and duration of exposure were recorded.

### Outcome

The AEDC is a standardized assessment performed across the country every 3 years by a child’s school teacher during the first year of full-time school (at age 4-6 years).^17^ It is a validated assessment in this age group, with good concurrent validity and internal reliability.^18^ Only children who commenced school during 1 of the years the AEDC was performed (2015, 2018, or 2021) were eligible for linkage. Children were assessed across 5 domains of development: (1) physical health and well-being, (2) social competence, (3) emotional maturity, (4) school-based language and cognitive skills, and (5) communication skills and general knowledge.

Our primary outcome was developmental vulnerability, defined as scoring below the 10th percentile in 2 or more of the 5 AEDC domains. We also examined the association between metformin and childhood scores below the 10th percentile on each of the 5 individual developmental domains. Developmental vulnerability on the AEDC provides an important estimation of emotional and behavioral problems throughout primary school and literacy and numeracy delays in high school.^19^

### Exclusions

Children born with congenital anomalies identified by *International Statistical Classification of Diseases, Tenth Revision, Clinical Modification* codes Q00 to Q99 were excluded. We additionally excluded women with uncertain diabetes diagnoses (eg, unknown timing of diabetes onset, 2 concurrent diabetes diagnoses) and those prescribed insulin without a recorded diabetes diagnosis.

### Statistical Analysis

The data analysis was performed between March 14, 2025, and January 30, 2026. The association between metformin exposure and childhood developmental vulnerability was estimated using inverse probability of treatment weighting combined with regression adjustment to correct for both nonrandom exposure to metformin and postexposure covariate effects. Data are presented as RR and percentage risk differences with 95% CIs. Statistical significance was inferred if the 95% CI excluded the null (1.00 for relative measures and 0 for absolute measures), corresponding to a 2-sided α = .05. The data analysis was completed using StataMP, version 18 (StataCorp LLC) in accordance with a prespecified statistical analysis plan agreed upon by all coauthors prior to commencing the study (eAppendix in Supplement 1).

Demographic and clinical characteristics are presented as frequencies and percentages for categorical variables and as mean (SD) or median (IQR) for normally distributed and skewed continuous variables, respectively. The association between metformin exposure and childhood developmental vulnerability was estimated using inverse probability–weighted regression adjustment (IPWRA) and presented as RR and percentage risk differences.

#### Missing Data

We compared missing data across variables and stratified by metformin exposure status. Children who did not receive AEDC scores but were identified by their school as having special educational needs had outcome data deterministically imputed to represent developmental vulnerability across all 5 domains.^20,21^ Missing outcome data for children not identified as having special needs and missing covariate data were considered missing at random and were imputed using multiple imputation with chained equations.

To obtain compatibility between the imputation process and analysis, we used the von Hippel-Bartlett approach in which 500 bootstrap resamples were first generated, with 2 imputations performed within each bootstrap sample, resulting in 1000 completed sets.^22^ Patterns of imputed data were compared with those in the original dataset to assess imputation adequacy, with no material differences identified. There were no missing exposure data.

#### IPWRA

In brief, IPWRA involves the creation of a propensity score based on covariates identified a priori as influencing metformin prescription during pregnancy, informed by a directed acyclic graph generated by our expert authorship team (eFigures 1 and 2 in Supplement 1). These covariates included maternal age, body mass index (BMI) (calculated as weight in kilograms divided by height in meters squared), maternal education, Socio-Economic Indexes for Areas, diabetes in pregnancy, and use of assisted reproductive technology (ART). The propensity score specifications included main-effects terms for ART and diabetes and an interaction between them modeled using a fully saturated set of exposure indicators representing all ART-by-diabetes combinations. This specification improved covariate balance compared with additive main-effects models and was therefore used in the final analysis. Not all variables specified in the causal framework were available in the dataset. Potential proxies (eg, country of birth for race and ethnicity) were considered but not included as evidence suggested poor validity.^23^

Propensity scores were inversely weighted, and postweighting covariate balance was assessed using absolute standardized mean differences (SMDs) within each of the 1000 imputed and bootstrapped datasets. The absolute SMDs and their corresponding standard deviations were then calculated across all datasets (Figure 1; eTable 2 in Supplement 1). An SMD of less than 0.1 was considered acceptable.^24^ There was complete global overlap of propensity scores across the datasets. To account for clustering of multiple births per woman, standard errors were adjusted using a unique maternal identifier.

**Figure 1.**
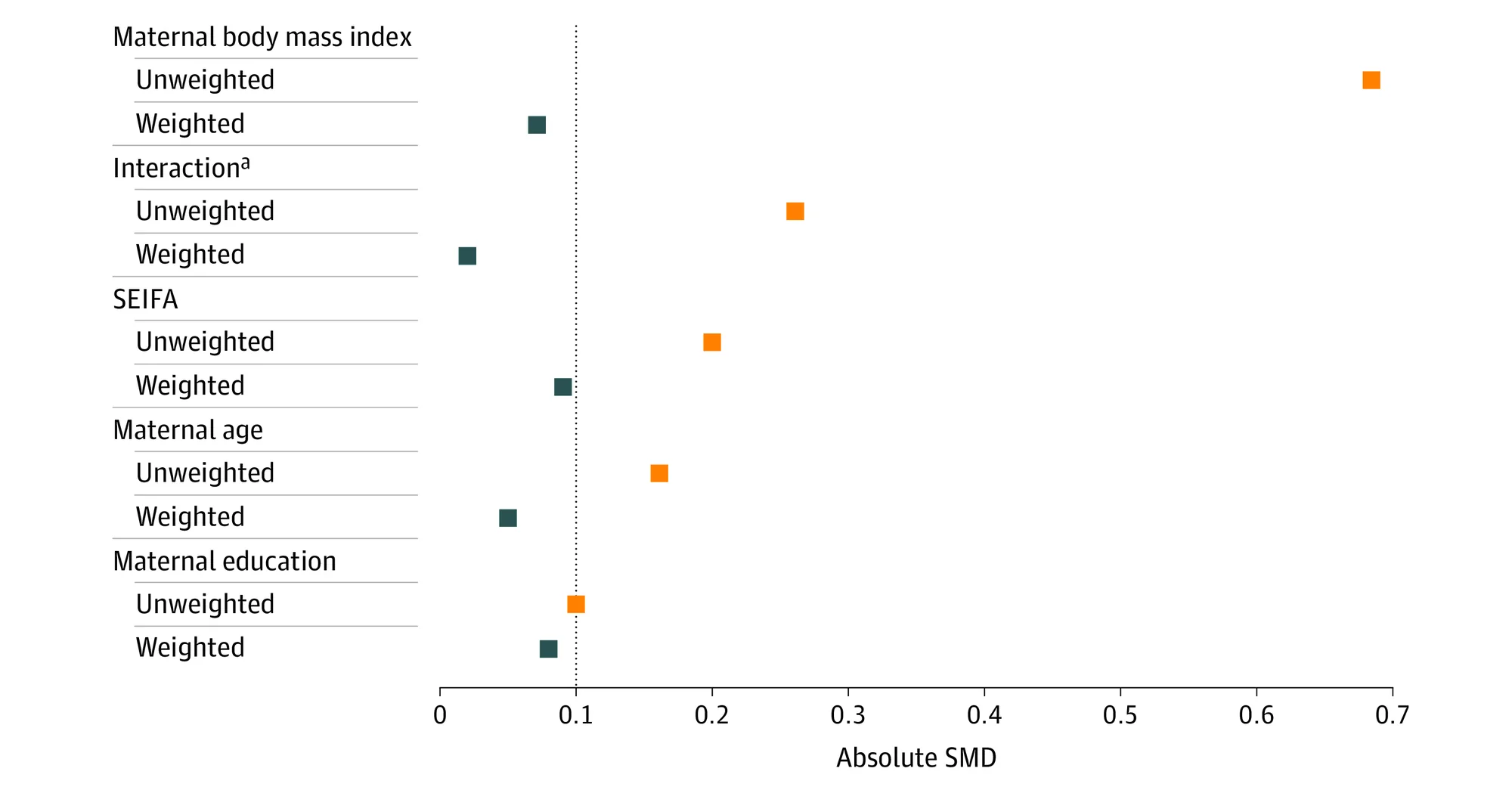
Dot Plot of the Propensity Score Covariate Balance for the Primary Cohort Propensity score covariate balance was assessed using standardized mean differences (SMDs) within each of the 1000 imputed datasets used in this study, with the average SMD across the imputed datasets presented. SEIFA indicates Socio-Economic Indexes for Areas. ^a^An interaction between diabetes in pregnancy and assisted reproductive therapy was introduced in the propensity score model after initial diagnostics indicated residual imbalance.

Inverse probability weighting was then combined with regression adjustment for preeclampsia, maternal education, language background other than English, Socio-Economic Indexes for Areas, birth weight, age at time of AEDC testing, year of AEDC, gestational age at birth, and child sex assigned at birth, chosen using prespecified direct acyclic graphs (eAppendix in Supplement 1). For each of the 1000 bootstrap-imputed datasets, an IPWRA model was fitted. Within each analysis, the propensity score was reestimated using the prespecified covariates, and inverse probability weights were generated and checked before estimation of the regression-adjusted treatment effect. The resulting treatment estimates were subsequently pooled using the von Hippel-Bartlett approach.^22^ A detailed description of this analysis technique is described in the eAppendix in Supplement 1.

#### Sensitivity Analyses

Metformin is most commonly prescribed during pregnancy for the management of diabetes.^25^ In light of the association between diabetes and other factors that may influence childhood development,^26^ we performed the analysis restricting the cohort to include only women with type 2 diabetes or gestational diabetes to eliminate the potential for confounding by indication. Early pregnancy is a critical period for fetal neurodevelopment,^27^ and we additionally examined whether first trimester metformin prescription was associated with an altered risk of overall childhood developmental vulnerability. Our primary analysis was repeated, excluding children with imputed outcome data, including those flagged as having special educational needs and those with incomplete outcome data for an unknown reason. Finally, alternative analytic approaches were explored. Regression adjustment analyses including postexposure variables were performed across 1000 bootstraps and imputations, with results again pooled using the von Hippel-Bartlett approach. A complete-case multivariable logistic regression using the same covariates was conducted as a sensitivity analysis for the primary IPWRA model, estimating the direct effect of exposure. Inverse probability weighting alone was performed across the same 1000 datasets to estimate the total effect.

## Results

### Demographic and Clinical Characteristics

From 871 627 singleton births in Victoria between 2009 and 2020, we identified 177 409 children with linked developmental outcome data, of whom 1095 (0.6%) were exposed to metformin antenatally (43 [3.9%] aged ≤5 years 0 months, 860 [78.5%] aged 5 years 1 month to 6 years 0 months, and 192 [17.5%] aged ≥6 years 1 month at assessment; 557 male [50.9%] and data suppressed for female to not unmask the number of children with missing sex) (Figure 2; Table 1). The 1095 mothers exposed to metformin during pregnancy were a mean (SD) age at delivery of 31.3 (5.3) years, and 309 (28.2%) were born outside Australia. Metformin exposure was common in the first trimester (703 women [64.2%]), with 141 women (12.9%) commencing metformin in the second trimester and 251 (22.9%) commencing metformin in the third trimester. A total of 467 women (42.6%) were dispensed more than 1 metformin prescription during pregnancy.

**Figure 2.**
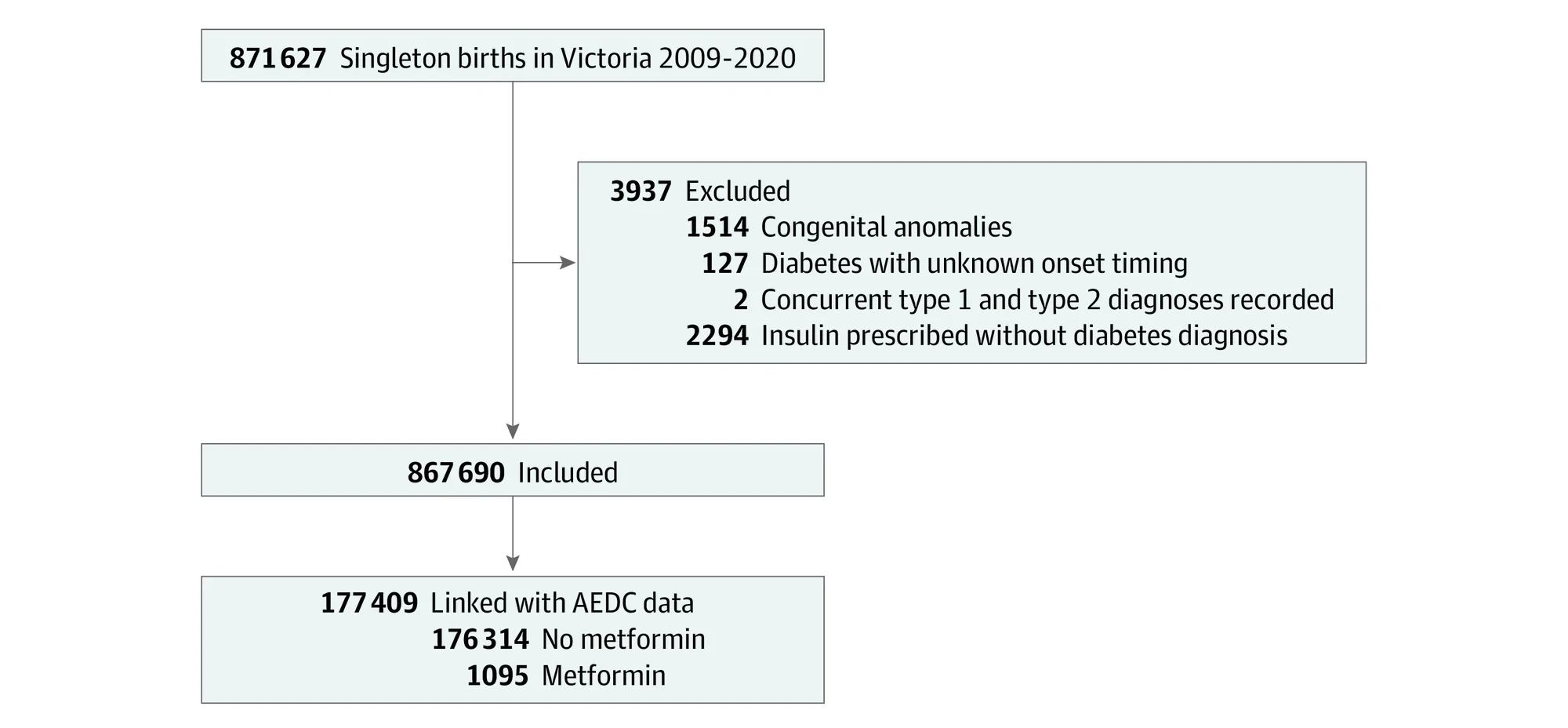
Participant Flow Diagram AEDC indicates Australian Early Development Census.

**Table 1.** Demographic and Clinical Characteristics by Metformin Exposure

Characteristic	Individuals, No. (%)	
	Not exposed to metformin (n = 176 314)	Exposed to metformin (n = 1095)
**Maternal**		
Age at delivery, mean (SD), y	31.3 (5.4)	32.2 (5.1)
Missing	0	0
BMI, mean (SD)	26.0 (5.8)	32.3 (9.1)
Missing	13 997 (7.9)	55 (5.0)
Parity		
Nulliparous	77 402 (43.9)	482 (44.0)
Multiparous	98 878 (56.1)	613 (56.0)
Missing	34 (<0.1)	0
Born outside Australia	49 063 (27.8)	309 (28.2)
Missing	5450 (3.1)	35 (3.2)
SEIFA quintile		
1 (Most deprivation)	27 996 (15.9)	231 (21.1)
2	31 951 (18.1)	239 (21.8)
3	39 625 (22.5)	233 (21.3)
4	41 349 (23.4)	237 (21.6)
5 (Least deprivation)	35 187 (19.9)	155 (14.2)
Missing	206 (0.1)	0
Highest education level		
Year ≤10	10 441 (5.9)	83 (7.6)
High school	20 586 (11.7)	114 (10.4)
Trade, TAFE, diploma, or certificate	67 653 (38.4)	470 (42.9)
Undergraduate university degree or higher	70 453 (40.0)	384 (35.1)
Missing	7181 (4.1)	44 (4.0)
Comorbidities		
Polycystic ovary syndrome	4086 (2.3)	250 (22.8)
Preeclampsia	4341 (2.5)	66 (6.0)
Gestational diabetes	14 620 (8.3)	429 (39.2)
Type 1 diabetes	175 (0.1)	10 (0.9)
Type 2 diabetes	876 (0.5)	196 (17.9)
Assisted reproductive technology (yes)	7559 (4.3)	128 (11.7)
Missing	599 (0.3)	DS (<0.5)
**Child**		
Sex assigned at birth		
Female	86 147 (48.9)	NA^a^
Male	90 022 (51.1)	557 (50.9)
Missing	145 (0.1)	DS (<0.1)
Gestational age at birth, median (IQR), wk	39.6 (38.6-40.4)	38.6 (37.9-39.6)
Missing	599 (0.3)	0
Preterm birth (yes)	10 419 (5.9)	137 (12.5)
Missing	599 (0.3)	0
Mode of birth		
Unassisted vaginal	93 954 (53.3)	440 (40.2)
Assisted vaginal	25 955 (14.7)	119 (10.9)
Emergency caesarean delivery	26 707 (15.2)	277 (25.3)
Elective caesarean delivery	29 623 (16.8)	259 (23.7)
Missing	75 (<0.1)	0
Birth weight, mean (SD), g	3404 (533)	3336 (594)
Missing	853 (0.5)	13 (1.1)
Birth weight >97th percentile^b^	3261 (1.9)	69 (6.3)
Missing	1451 (0.8)	13 (1.2)
Birth weight <10th percentile^b^	19 328 (11.0)	113 (10.3)
Missing	1451 (0.8)	13 (1.2)
Language background other than English	35 985 (20.4)	242 (22.1)
Missing	0	0
Age at assessment		
≤5 y 0 mo	2861 (1.6)	43 (3.9)
5 y 1 mo to 6 y 0 mo	138 944 (78.8)	860 (78.5)
≥6 y 1 mo	34 509 (19.6)	192 (17.5)
Missing	0	0
Special educational needs	8980 (5.1)	82 (7.5)
Incomplete AEDC data	1036 (0.6)	DS (<0.5)

Abbreviations: AEDC, Australian Early Development Census; BMI, body mass index (calculated as weight in kilograms divided by height in meters squared); DS, data suppressed due to small numbers; NA, not available; SEIFA, Socio-Economic Indexes for Areas; TAFE, Technical and Further Education.

^a^ Number of female children not reported per the data custodian as it would unmask the number of children with missing sex.

^b^ Hadlock percentiles.

Demographic and clinical characteristics were broadly comparable between children with and without linked developmental data. However, women with linked data compared with those without were less commonly born outside Australia (49 372 [27.8%] vs 239 608 [34.7%]) and had a lower incidence of gestational diabetes (15 049 [8.5%] vs 80 085 [11.6%]) (eTable 3 in Supplement 1).

No clear pattern of missingness was observed. Specifically, BMI missingness was 7.9% and 8.2% for developmental vulnerability and nonvulnerability, respectively (*P* = .33).

Women using metformin had a higher mean (SD) BMI of 32.3 (9.1) compared with women not using metformin (26.0 [5.8]) (Table 1). A higher proportion of women prescribed metformin conceived via ART (128 [11.7%] vs 7559 [4.3%]) and more commonly had comorbidities, including polycystic ovary syndrome (250 [22.8%] vs 4086 [2.3%]), type 2 diabetes (196 [17.9%] vs 876 [0.5%]), and gestational diabetes (429 [39.2%] vs 14 620 [8.3%]).

Women exposed to metformin compared with those not exposed more commonly gave birth pre term (<37 weeks’ gestation) (137 [12.5%] vs 10 419 [5.9%]) and by caesarean delivery (536 [49.0%] vs 56 330 [32.0%]). Infants exposed to metformin more commonly had birth weights above the 97th percentile compared with those not exposed (69 [6.3%] vs 3261 [1.9%]). The proportion of infants with birth weights below the 10th percentile was similar between groups (113 [10.3%] vs 19 328 [11.0%]).

### Developmental Vulnerability

Among children exposed to metformin in utero, 199 (18.2%) were considered developmentally vulnerable, scoring below the 10th percentile across at least 2 of the 5 assessed domains in the AEDC (Table 2) compared with 24 379 children (13.9%) without exposure. This finding includes 9062 children (5.1%) across the cohort who were considered as having special educational needs (Table 1).

**Table 2.** Developmental Outcomes on the AEDC by Metformin Exposure

Domain	Individuals, No. (%)		Unadjusted effect estimate		Adjusted effect estimate using IPWRA^a^	
	Not exposed to metformin (n = 176 314)	Exposed to metformin (n = 1095)	RR (95% CI)	% RD (95% CI)	RR (95% CI)	% RD (95% CI)
Developmental vulnerability (scoring <10th percentile in ≥2 domains)	24 379 (13.9)	199 (18.2)	1.31 (1.16 to 1.49)	4.32 (2.04 to 6.62)	0.97 (0.74 to 1.29)	−0.23 (−4.01 to 3.54)
Missing	443 (1.8)	DS (<1.0)				
Domain-specific developmental vulnerability						
Physical health and well-being	21 898 (12.4)	196 (17.9)	1.44 (1.27 to 1.63)	5.46 (3.18 to 7.73)	0.98 (0.76 to 1.27)	−0.13 (−3.31 to 3.04)
Missing	297 (1.3)	0				
Social competence	22 932 (13.0)	190 (17.4)	1.33 (1.17 to 1.52)	4.32 (2.07 to 6.57)	1.00 (0.74 to 1.36)	0.21 (−3.71 to 4.12)
Missing	303 (1.3)	0				
Emotional maturity	21 798 (12.4)	167 (15.3)	1.23 (1.07 to 1.42)	2.87 (0.73 to 5.01)	0.84 (0.64 to 1.10)	−1.94 (−4.74 to 0.87)
Missing	895 (3.9)	DS (<5.0)				
Language and cognitive skills (school based)	18 828 (10.7)	173 (15.8)	1.48 (1.29 to 1.69)	5.11 (2.94 to 7.28)	1.09 (0.82 to 1.45)	1.08 (−2.28 to 4.44)
Missing	419 (2.2)	DS (<1.0)				
Communication skills and general knowledge	19 141 (10.9)	164 (15.0)	1.38 (1.20 to 1.59)	4.10 (1.98 to 6.22)	0.90 (0.68 to 1.19)	−1.00 (−3.73 to 1.73)
Missing	308 (1.6)	0				

Abbreviations: % RD, percentage risk difference; AEDC, Australian Early Development Census; DS, data suppressed due to small numbers; IPWRA, inverse probability–weighted regression adjustment; RR, relative risk; SEIFA, Socio-Economic Indexes of Areas.

^a^ Selection model covariates included maternal age, maternal body mass index, maternal education, SEIFA, diabetes in pregnancy, and use of assisted reproductive technology. Outcome model covariates included preeclampsia, maternal education, language background other than English, SEIFA, birth weight, age at time of AEDC testing, year of AEDC, gestational age at birth, and child sex assigned at birth.

For the association between antenatal metformin exposure and overall developmental vulnerability at school entry, the unadjusted RR was 1.31 (95% CI, 1.16-1.49). However, following adjustment via doubly robust inverse probability weighting, this association was no longer observed (adjusted RR [ARR], 0.97 [95% CI, 0.74-1.29]). For each of the 5 domains of the AEDC individually, we found that metformin was also not associated with an altered risk of scoring below the 10th percentile in any developmental domains (physical health and well-being: ARR, 0.98 [95% CI, 0.76-1.27]; social competence: ARR, 1.00 [95% CI, 0.74-1.36]; emotional maturity: ARR, 0.84 [95% CI, 0.64-1.10]; language and cognitive skills [school-based]: ARR, 1.09 [95% CI, 0.82-1.45]; communication skills and general knowledge: ARR, 0.90 [95% CI, 0.68–1.19]) (Table 2).

### Sensitivity Analyses

The most common indication for metformin in pregnancy is diabetes.^25^ To eliminate diabetes as a confounder, we restricted the cohort to include only women with gestational diabetes or type 2 diabetes (n = 16 121). In this group, there were 129 children (20.7%) exposed to metformin considered developmentally vulnerable compared with 2460 children (15.9%) without exposure. Unadjusted, metformin was associated with an increased risk of developmental vulnerability (RR, 1.30 [95% CI, 1.11-1.52]). However, after adjustment for confounders, no association was observed (ARR, 1.08 [95% CI, 0.86-1.35]) (Table 3).

**Table 3.** Developmental Outcomes on the AEDC by Metformin Exposure Among Women With Gestational Diabetes or Type 2 Diabetes in Pregnancy

Domain	Women, No. (%)		Unadjusted effect estimate		Adjusted effect estimate using IPWRA^a^	
	Not exposed to metformin (n = 15 496)	Exposed to metformin (n = 625)	RR (95% CI)	% RD (95% CI)	RR (95% CI)	% RD (95% CI)
Developmental vulnerability (scoring <10th percentile in ≥2 domains)	2460 (15.9)	129 (20.7)	1.30 (1.11 to 1.52)	4.75 (1.52 to 7.98)	1.08 (0.86 to 1.35)	1.35 (−2.53 to 5.23)
Missing	43 (0.3)	DS (<1.0)				
Domain-specific developmental vulnerability						
Physical health and well-being	2183 (14.1)	125 (20.0)	1.41 (1.20 to 1.66)	5.84 (2.68 to 9.00)	1.11 (0.88 to 1.39)	1.65 (−2.01 to 5.31)
Missing	33 (0.2)	0				
Social competence	2260 (14.6)	123 (19.7)	1.37 (1.16 to 1.60)	5.37 (2.21 to 8.53)	1.16 (0.90 to 1.50)	2.54 (−1.63 to 6.71)
Missing	30 (0.2)	0				
Emotional maturity	2089 (13.6)	103 (16.6)	1.23 (1.03 to 1.47)	3.18 (0.22 to 6.14)	0.94 (0.74 to 1.20)	−0.70 (−3.82 to 2.42)
Missing	90 (0.6)	DS (<1.0)				
Language and cognitive skills (school based)	1906 (12.3)	103 (16.5)	1.37 (1.15 to 1.64)	4.55 (1.59 to 7.52)	1.03 (0.80 to 1.33)	0.48 (−2.73 to 3.71)
Missing	41 (0.3)	DS (<1.0)				
Communication skills and general knowledge	2079 (13.4)	108 (17.3)	1.30 (1.09 to 1.55)	4.03 (1.03 to 7.04)	1.07 (0.84 to 1.36)	1.04 (−2.47 to 4.55)
Missing	32 (0.2)	0				

Abbreviations: % RD, percentage risk difference; AEDC, Australian Early Development Census; DS, data suppressed due to small numbers; IPWRA, inverse probability–weighted regression adjustment; RR, relative risk; SEIFA, Socio-Economic Indexes of Areas.

^a^ Selection model covariates included maternal age, maternal body mass index, maternal education, SEIFA, diabetes in pregnancy, and use of assisted reproductive technology. Outcome model covariates included preeclampsia, maternal education, language background other than English, SEIFA, birth weight, age at time of AEDC testing, year of AEDC, gestational age at birth, and child sex assigned at birth.

Similarly, investigating each of the 5 domains of the AEDC individually in this cohort with gestational diabetes or type 2 diabetes, metformin was not associated with an altered risk of scoring below the 10th percentile in any developmental domain (physical health and well-being: ARR, 1.11 [95% CI, 0.88-1.39]; social competence: ARR, 1.16 [95% CI, 0.90-1.50]; emotional maturity: ARR, 0.94 [95% CI, 0.74-1.20]; language and cognitive skills [school-based]: ARR, 1.03 [95% CI, 0.80-1.33]; communication skills and general knowledge: ARR, 1.07 [95% CI, 0.84-1.36]) (Table 3).

We performed the same analysis while considering only children with complete outcome data and excluding children with special educational needs (9062 [5.1%]). We observed that 117 children (11.6%) exposed to metformin were considered developmentally vulnerable compared with 15 399 children (9.2%) not exposed. After adjustment for confounders, we observed no association between metformin exposure and developmental risk in this group (ARR, 0.80 [95% CI, 0.53-1.23]) (eTable 4 in Supplement 1).

Considering whether first trimester exposure was associated with developmental outcomes, 121 children (17.2%) with first trimester metformin exposure were considered developmentally vulnerable compared with 24 379 children (13.9%) not exposed. After adjustment for covariates, we observed no association between metformin exposure and developmental vulnerability in this cohort (ARR, 1.07 [95% CI, 0.77-1.48]) (eTable 4 in Supplement 1). Finally, results were robust to alternative specifications of the model (eTable 5 in Supplement 1).

## Discussion

This cohort study of 177 409 children in Victoria, Australia, found no association between metformin prescription in pregnancy and developmental vulnerability in the first year of full-time school. No association was observed across all assessed developmental domains from the AEDC and with restriction of the cohort to include only women with gestational diabetes or type 2 diabetes in pregnancy.

To date, there have only been 2 other observational studies that examined the association of metformin use in pregnancy with childhood development.^28,29^ The first included a cohort of 1681 children born to women with gestational diabetes, which observed no association between metformin exposure and teacher-assessed offspring behavioral difficulties prior to school entry.^28^ Our analysis supports this finding in an older group of children both after restricting the cohort to include only women with gestational diabetes or type 2 diabetes and in our primary analysis, which included children whose mothers were prescribed metformin for any indication. This finding is particularly relevant given the off-label prescription of metformin for nondiabetes indications, including treatment for polycystic ovary syndrome and obesity.^30,31^

The second study observed no association between metformin exposure and challenges in offspring motor-social development at various ages (2114 children exposed to metformin at age 3-5 years, 899 at age 6-8 years, and 290 at age 9-11 years) based on *International Statistical Classification of Diseases, Tenth Revision* codes.^29^ Our findings are also consistent with this study, though our study considers development across a range of domains rather than by diagnostic criteria. Children from lower socioeconomic backgrounds are less able to access and receive developmental diagnoses,^32,33^ and using a nationally performed assessment tool, as we have done, ensures that these children are not misclassified as developmentally on track.

The 5 randomized clinical trials in this area were small and typically used short follow-up (eg, into infancy) but, reassuringly, also demonstrated no association between metformin use and adverse offspring neurodevelopment.^34,35,36,37,38^ Overall, our study is in keeping with current research observing no association between in utero metformin exposure and adverse childhood neurodevelopment.

### Strengths and Limitations

The strength of this study lies in the large number of children exposed antenatally to metformin followed up to the first year of full-time school. We used the NDSS enriched with the VPDC to identify a comprehensive cohort of women with diabetes in pregnancy. Furthermore, we considered childhood development across a range of physical and intellectual domains using a national and validated assessment tool. We used a doubly robust estimator combined with multiple imputation and bootstrapping to address some of the bias typically associated with observational data. Indication for metformin was not directly available in our study, though maternal comorbidities provided insight into the potential reason for metformin prescription and were included as covariates.

This study also had some limitations. There remains the potential for residual confounding, including by race and ethnicity. Self-reported race and ethnicity data were not available, and while maternal country of birth was considered as a potential proxy, it was not included in the final models due to high heterogeneity and limited validity as a measure of race and ethnicity in an Australian population.^23^ Furthermore, while residual confounding could not be entirely excluded, its influence would have been expected to bias estimates toward a seemingly harmful association. The absence of an association after adjustment is hence particularly notable and reassuring.

Another limitation is that we were unable to assess medication adherence. However, we considered women as exposed to metformin only if they had been dispensed the medication from a pharmacy, which provides a more important estimation of adherence than medication prescription alone. There were 467 women (42.6%) who were dispensed more than 1 metformin prescription during pregnancy, and despite the large cohort, planned sensitivity analyses investigating the association of multiple prescriptions and second and third trimester metformin commencement were underpowered to support doubly robust or fully adjusted models. A small number of women may have been dispensed a metformin prescription immediately prior to conception and continued treatment into early pregnancy without requiring an additional dispensing during pregnancy. As our exposure definition relied on dispensing records during pregnancy, these women would have been misclassified as not exposed to metformin. In this instance though, the duration of unrecorded exposure may have been short and therefore may not have materially influenced longer-term offspring outcomes. Similarly, we could not ascertain metformin dose from our dataset. With the rising prescription of metformin during pregnancy,^2^ future studies may be better placed to investigate this.

## Conclusions

This cohort study found no evidence between in utero metformin exposure and developmental vulnerability in early primary school–aged children. Alongside previously published research in this area, the finding may provide reassurance for clinicians and women considering metformin use during pregnancy. Further research is still needed to examine the association of antenatal metformin use with offspring growth and cardiometabolic health, with current research in this area conflicting.^9,10,39^

## Notes

Supplement 1. **eTable 1.** Methods Used to Ascertain Clinical Characteristics **eTable 2.** Propensity Score Covariate Balance Assessment **eTable 3.** Demographic and Clinical Characteristics for Participants With and Without Linked Developmental Outcome Data **eTable 4.** Sensitivity Analyses: Childhood Developmental Vulnerability on the Australian Early Development Census by Subgroup **eTable 5.** Sensitivity Analyses: Childhood Developmental Vulnerability on the Australian Early Development Census by Analysis Type **eAppendix.** Does In Utero Exposure to Metformin Alter the Risk of Childhood Neurodevelopment at School Entry? A Statistical Analysis Plan **eFigure 1.** Directed Acyclic Graph: Metformin in Pregnancy and Offspring Development **eFigure 2.** Directed Acyclic Graph: Sensitivity Analysis—Metformin in Pregnancy Among Women With Diabetes (Type 2, Gestational Diabetes) and Offspring Development
eReferences.

Supplement 2.
Data Sharing Statement
