# Supplementary material for: Metformin Exposure in Pregnancy and Childhood Developmental Outcomes
Source: JAMA Netw Open. 2026 Sep 30;9(9):e2637054. doi: 10.1001/jamanetworkopen.2026.37054 (PMC13628313; doi:10.1001/jamanetworkopen.2026.37054)
Supplement: Supplementary file 1 [file jamanetwopen-e2637054-s001.pdf]

Supplemental Online Content

Gordon H, Hiscock RJ, Price S, et al. Metformin exposure in pregnancy and childhood developmental outcomes. *JAMA Netw Open*. 2026;9(9):e2637054.
doi:10.1001/jamanetworkopen.2026.37054

eTable 1. Methods Used to Ascertain Clinical Characteristics

eTable 2. Propensity Score Covariate Balance Assessment

eTable 3. Demographic and Clinical Characteristics for Participants With and Without Linked Developmental Outcome Data

eTable 4. Sensitivity Analyses: Childhood Developmental Vulnerability on the Australian Early Development Census by Subgroup

eTable 5. Sensitivity Analyses: Childhood Developmental Vulnerability on the Australian Early Development Census by Analysis Type

eAppendix. Does In Utero Exposure to Metformin Alter the Risk of Childhood Neurodevelopment at School Entry? A Statistical Analysis Plan

eFigure 1. Directed Acyclic Graph: Metformin in Pregnancy and Offspring Development

eFigure 2. Directed Acyclic Graph: Sensitivity Analysis—Metformin in Pregnancy Among Women With Diabetes (Type 2, Gestational Diabetes) and Offspring Development

eReferences

This supplemental material has been provided by the authors to give readers additional information about their work.

© 2026 Gordon H et al. *JAMA Network Open*.

eTable 1. Methods Used to Ascertain Clinical Characteristics

Clinical characteristic	Source	Description
Gestational diabetes	Victorian Perinatal Data Collection and/or National Diabetes Service Scheme	IDC code O244 in the Victorian Perinatal Data Collection included as a maternal medical condition, indication for induction of labour, or a labour or postpartum complication and/or a recorded diagnosis of gestational diabetes in the National Diabetes Service Scheme during pregnancy
Type 1 diabetes	National Diabetes Service Scheme, Pharmaceutical Benefits Scheme	Recorded diagnosis of Type 1 diabetes prior to or during pregnancy in the National Diabetes Service Scheme, with an insulin prescription during pregnancy or within 90 days preconception
Type 2 diabetes	National Diabetes Service Scheme	Recorded diagnosis of Type 2 diabetes prior to or during pregnancy
Pregnancy conceived using artificial reproductive technology	Victorian Perinatal Data Collection and/or Victorian Admitted Episodes Dataset	1) Coded "yes" as checkbox response to "Was this pregnancy conceived via artificial reproductive technology in the Victorian Perinatal Data Collection; or<br>2) Having a hospital admission in the Victorian Admitted Episodes Dataset with ICD codes: Z31.1 (Artificial insemination), Z31.2 (In vitro fertilisation), Z31.3 (Other assisted fertilisation methods) or N98 (Complications associated with ART); or<br>3) With a procedure during hospital admission in the Victorian Admitted Episodes Dataset including gamete intrafallopian transfer, ovulation monitoring for artificial insemination, fresh, frozen or thawed embryo transfer to uterus or fallopian tube within 30 days prior to first day of current pregnancy
Preeclampsia	Victorian Perinatal Data Collection	IDC codes O11, O14.0-O14.9 or O15.0-O15.9 included as a maternal medical condition, indication for induction of labour, or a labour or postpartum complication.
Polycystic ovary syndrome	Victorian Perinatal Data Collection	ICD code E28.2 included as a maternal medical condition, indication for induction of labour, or a labour or postpartum complication.

Abbreviation: ICD = International Classification of Diseases 10

© 2026 Gordon H et al. *JAMA Network Open*.

eTable 2. Propensity Score Covariate Balance Assessment[1]

Covariate	Raw absolute SMD (SD)	Post-IPW absolute SMD (SD)
**Primary analysis**		
Maternal body mass index	0.84 (0.03)	0.08 (0.03)
Interaction[2]	0.26 (0.02)	0.02 (0.01)
Socio-economic indexes for areas	0.20 (0.03)	0.09 (0.03)
Maternal age	0.16 (0.03)	0.05 (0.03)
Maternal education	0.10 (0.03)	0.08 (0.03)
**Sensitivity analysis: Diabetes-only cohort**		
Maternal body mass index	0.62 (0.04)	0.03 (0.02)
Conception with artificial reproductive technology	0.09 (0.04)	0.03 (0.02)
Socio-economic indexes for areas	0.21 (0.04)	0.04 (0.03)
Maternal age	0.04 (0.03)	0.02 (0.02)
Maternal education	0.24 (0.04)	0.03 (0.02)

Abbreviations: SMD = Standardised mean difference; SD = Standard deviation; IPW = Inverse probability weighting.
[1]Propensity score covariate balance was assessed using absolute SMDs within each of the imputed datasets. The table presents the average absolute SMDs across the 1000 datasets, with the corresponding SD. [2]An interaction between diabetes in pregnancy and ART was introduced in the propensity score model after initial diagnostics indicated residual imbalance.

© 2026 Gordon H et al. *JAMA Network Open*.

eTable 3. Demographic and Clinical Characteristics for Participants With and Without Linked Developmental Outcome Data

	**Linked data N=177,409 (n, %)**	**Unlinked data N=690,281 (n, %)**
**Maternal clinical and demographic characteristics**		
**Maternal age at birth**, Mean (SD)<br>Missing	31.3 (5.3)<br>0 (0)	31.5 (5.3)<br>58 (<0.1)
**Body mass index**, Mean (SD)<br>Missing	26.0 (5.9)<br>14,044 (7.9)	26.0 (5.9)<br>40.354 (5.8)
**Parity**<br>-Nulliparous<br>-Multiparous<br>-Missing	 77,884 (43.9)<br>99,491 (56.1)<br>34 (<0.1)	 306,833 (44.5)<br>383,009 (55.5)<br>439 (0.1)
**Born outside Australia (yes)**<br>Missing	49,372 (27.8)<br>5,485 (3.1)	239,608 (34.7)<br>20,554 (3.0)
**Maternal comorbidities**<br>- Polycystic ovary syndrome<br>- Preeclampsia<br>- Gestational diabetes<br>- Type 1 diabetes<br>- Type 2 diabetes	 4,336 (2.4)<br>4,407 (2.5)<br>15,049 (8.5)<br>185 (0.1)<br>1,072 (0.6)	 20,397 (3.0)<br>17,239 (2.5)<br>80,085 (11.6)<br>923 (0.1)<br>4,282 (0.6)
**Assisted reproductive technology (yes)**<br>Missing	7,687 (4.3)<br>599 (0.3)	31,775 (4.6)<br>3,856 (0.6)
**Child clinical and demographic characteristics**		
**Sex at birth, male**<br>Missing	90,579 (51.1)<br>DS (0.1)	354,387 (51.3)<br>395 (0.1)
**Gestational age at birth** (weeks), Median (IQR)<br>Missing	39.6 (38.6, 40.4)	39.3 (38.4, 40.3)
**Preterm birth (yes)**<br>Missing	10,556 (6.0)<br>DS (<0.5)	45,561 (6.6)<br>2,211 (0.3)
**Mode of birth**<br>- Unassisted vaginal birth<br>- Assisted vaginal birth<br>- Emergency caesarean section<br>- Elective caesarean section<br>- Missing	 94,394 (53.2)<br>26,074 (14.7)<br>26,984 (15.2)<br>29,882 (16.8)<br>75 (<0.1)	 352,322 (51.0)<br>105,729 (15.3)<br>112,624 (16.3)<br>119,513 (17.3)<br>93 (<0.1)
**Birthweight (g),** Mean (SD)<br>Missing	3403 (533)	3359 (565)
**Birthweight >97th centile**[1]<br>Missing	3,330 (1.9)<br>1,464 (0.8)	12,482 (1.8)<br>5,054 (0.7)
**Birthweight <10th centile**[1]<br>Missing	19,441 (11.0)<br>1,464 (0.8)	78,325 (11.3)<br>5,052 (0.7)

© 2026 Gordon H et al. *JAMA Network Open*.

eTable 4. Sensitivity Analyses: Childhood Developmental Vulnerability on the Australian Early Development Census (AEDC) by Subgroup

		Metformin unexposed	Metformin exposed	Unadjusted effect estimate (95% CI)		Adjusted effect estimate using IPWRA (95% CI)*	
				Relative risk	% Risk difference	Relative risk	% Risk difference
**Metformin exposure from first trimester**	**Total**	**N=175,871**	**N=702**				
	Developmental vulnerability (scoring <10th centile in ≥2 domains) (n, %)	24,379 (13.9)	121 (17.2)	1.24 (1.06, 1.46)	3.37 (0.58, 6.17)	1.07 (0.77, 1.48)	1.22 (-3.57, 6.01)
**Cohort restricted to children with complete outcome data**	**Total**	**N=167,058**	**N=896**				
	Developmental vulnerability (scoring <10th centile in ≥2 domains) (n, %)	15,399 (9.2)	117 (11.6)	1.25 (1.06, 1.49)	2.33 (0.36, 4.31)	0.80 (0.53, 1.23)	-1.62 (-4.86, 1.62)

Abbreviations: CI = Confidence interval, IPWRA = Inverse Probability Weighted Regression Adjustment. *Selection model covariates: Maternal age, maternal body mass index, maternal education, socioeconomic indexes for areas (SEIFA), and use of assisted reproductive technology (ART). Outcome model covariates: Preeclampsia, maternal education, language background other than English, SEIFA, birthweight, age at time of AEDC testing, year of AEDC, gestational age at birth and child sex at birth.

© 2026 Gordon H et al. *JAMA Network Open*.

eTable 5. Sensitivity Analyses: Childhood Developmental Vulnerability on the Australian Early Development Census (AEDC) by Analysis Type

	Unadjusted relative risk (95% CI)	Odds ratio (95% CI) using multivariate logistic regression[1]	Relative risk (95% CI) using regression adjustment[2]	Relative risk (95% CI) using inverse probability weighting[3]
Developmental vulnerability (scoring <10th centile in ≥2 domains)	1.31 (1.16, 1.49)	1.08 (0.90, 1.28)	0.99 (0.82, 1.19)	0.98 (0.81, 1.17)

Abbreviation: CI = Confidence interval. [1]Complete case analysis. Adjusted for maternal age, maternal body mass index (BMI), maternal education, socioeconomic indexes for areas (SEIFA), diabetes in pregnancy, use of assisted reproductive technology (ART), maternal preeclampsia, maternal education, language background other than English, age at time of AEDC testing, gestational age at birth and child sex at birth. [2]Adjusted for same variables as multivariate logistic regression analysis, results pooled across 1000 bootstraps and imputations. [3]Selection model covariates including maternal age, maternal BMI, maternal education, SEIFA, and use of ART. Results pooled across 1000 bootstraps and imputations.

© 2026 Gordon H et al. *JAMA Network Open*.

eAppendix. Does In Utero Exposure to Metformin Alter the Risk of Childhood Neurodevelopment at School Entry? A Statistical Analysis Plan

Version 3. 13th March 2025

Research team
Dr Hannah Gordon, PhD candidate (MD)[1,2]
Dr Richard Hiscock, Statistician and anaesthetist (MBBS)[1,2]
Dr Sarah Price, Obstetric medicine physician and endocrinologist (PhD)[1,3]
A/Prof Alexis Shub, Maternal fetal medicine specialist (PhD)[1,2]
Ms Alexandra Roddy-Mitchell, PhD candidate (MPH)[1,2]
Ms Anna Forsythe, Data analyst (MEnv)[1,2]
Ms Jessica Atkinson, PhD candidate (BBiomed) [1,2]
Professor Susan Walker, Maternal fetal medicine specialist (MD)[1,2]
Professor Stephen Tong, Clinician scientist and obstetrician (PhD)[1,2]
A/Professor Roxanne Hastie, Perinatal epidemiologist (PhD)[1,2]
Dr Anthea Lindquist, Perinatal epidemiologist and obstetrician (Dphil)[1,2]

[1]Department of Obstetrics, Gynaecology and Newborn Health, University of Melbourne, Parkville, Victoria, Australia
[2] Mercy Perinatal, Mercy Hospital for Women, Heidelberg, Victoria, Australia
[3] Royal Women's Hospital, Parkville, Victoria, Australia

Research question: What is the association between maternal metformin use in pregnancy and childhood developmental outcomes at school entry?

Population: All children born in Victoria, Australia between 2009 and 2021 with linked birth and early developmental outcome data.

Exposure: Maternal metformin prescription during pregnancy, as identified by the Pharmaceutical Benefits Scheme.

Comparator: Women without metformin prescription during pregnancy.

Primary outcome: Developmental vulnerability at school entry, assessed using the Australian Early Development Census and defined as scoring <10th centile in ≥2 domains.

Null hypothesis: Metformin use in pregnancy is not associated with an altered risk of developmental vulnerability at school entry.

1. Background

Metformin is an oral hypoglycaemic medication, first-line in the management of type 2 diabetes outside of pregnancy.[1] During pregnancy, metformin crosses the placenta, with the fetus exposed to similar medication concentrations as that in the maternal bloodstream.[2] In the context of reassuring short-term perinatal safety data,[3,4] the use of metformin in pregnancy is increasing.[5] However, the long-term safety for exposed offspring remains incompletely described.[6,7] Compared with insulin, children randomised to metformin exposure in pregnancy had similar metabolic profiles when followed up at 7-9 years of age,[8] but were marginally heavier, with higher waist-to-height ratios at 9 years of age, acknowledging that numbers were small (n=99).[8]

Beyond metabolic implications, in-utero metformin exposure has been shown to impair cortical development in animal models.[9] Reassuringly, no difference in cortical excitability was observed in metformin-exposed human offspring, compared with insulin.[10] Yet it is unclear whether metformin exposure translates to any clinically meaningful differences in neurodevelopment.

Our team recently performed a systematic review in this area, and identified seven studies; many of them small with variation in developmental assessment tools used and timing of follow-up.[7] Across these studies, no association between in-utero metformin exposure and global neurodevelopmental delay was found among children <2 years of age (RR 1.09 [95% CI: 0.54, 2.17]; three studies; 9668 children) and 3-5 years (RR 0.90 [95% CI: 0.56, 1.45]; 2 studies; 6118 children). Significant heterogeneity was observed between included studies,

© 2026 Gordon H et al. *JAMA Network Open*.

with differing initial indications for metformin (gestational diabetes; polycystic ovary syndrome; body mass index >25), and trimester of first exposure.

Sufficiently powered studies including outcomes for school-aged children (rather than toddlers) are therefore needed to provide sufficient long-term neurodevelopmental safety data for women and their care providers considering metformin use in pregnancy.

2. Study population

A linked cohort of 873,561 births from 2009-2021 across Victoria, Australia has been drawn from the following data sources:

- **Birth data:** the Victorian Perinatal Data Collection (VPDC) collects state-wide data relating to pregnancy, birth and perinatal outcomes, including maternal medical conditions, mode of delivery, gestational age at delivery and birthweight, and the presence of any congenital anomalies. It is mandatory for every birth to be recorded in the VPDC, with the database regularly audited to ensure data accuracy and completeness.[11]
- **Diabetes in pregnancy:** The National Diabetes Service Scheme (NDSS) is an Australian-wide register of patients with diabetes. The NDSS is the most comprehensive diabetes database in the country,[12] and registration to NDSS confers a subsidy of the cost of diabetes products like blood glucose monitoring strips, insulin pump consumables and insulin syringes. Each record is validated by an 8uthorized health professional to ensure accuracy. The NDSS will be used to ascertain accurate information about type and treatment of diabetes, and is estimated to capture 80-90% of people living with diabetes in Australia.[13]
- **Medication data:** the Pharmaceutical Benefit Scheme (PBS) provides information about medications dispensed and will be used to determine metformin prescription during pregnancy. Prescriptions must be filled by a pharmacy to be recorded in the PBS, with each metformin script typically resulting in the dispensing of 100 tablets. Insulin prescription will also be included to validate type 1 diabetes diagnoses in the cohort.
- **Early childhood development data:** the Australian Early Development Census (AEDC) is performed every three years nation-wide and assesses all children during the first year of fulltime school. Five domains are assessed: (1) Physical health and wellbeing, (2) Social competence, (3) Emotional maturity, (4) Language and cognitive skills (school-based), and (5) Communication skills and general knowledge. We will use AEDC data to ascertain overall childhood developmental vulnerability, and domain-specific vulnerability.[14]

Women with pre-existing diabetes or gestational diabetes will be identified using the NDSS, cross-checked against VPDC. Women with uncertain diabetes diagnoses will be excluded, including those with 1) unknown time of diabetes onset, 2) those prescribed insulin without an NDSS or VPDC diagnosis of diabetes (due to uncertain diabetes type) and 3) women with a recorded diagnosis of type 1 diabetes but without an insulin prescription during pregnancy or the three months preconception. Women without a diagnosis of diabetes in the NDSS or VPDC will not be considered as having diabetes in pregnancy.

Accordingly, we do not anticipate any missing exposure data.

Inclusion criteria:

- All children born in Victoria, Australia between 2009 and 2021 with linked AEDC outcome data.

Exclusion criteria:

- Children born with any congenital anomaly (ICD codes Q00-Q99)
- Multiple pregnancy
- Maternal insulin prescription without an NDSS or VPDC diagnosis of diabetes

Data linkage has been performed by the third-party Centre for Victorian Data Linkage (CVDL) using probabilistic determination. Data for analysis are de-identified.

3. Exposure

Women who have had one or more metformin scripts dispensed during pregnancy, from date of conception until date of birth. First day of last normal menstrual period will be calculated as the date of birth of the infant, minus the gestational age in days at time of birth.

4. Outcomes

Primary outcome: Childhood developmental vulnerability will be measured by AEDC scores below the 10th centile across two or more developmental domains.

© 2026 Gordon H et al. *JAMA Network Open*.

Secondary outcomes will include AEDC individual domain scores below the 10th centile across each of the five developmental domains (physical health and wellbeing, social competence, emotional maturity, language and cognitive skills (school-based), and communication skills and general knowledge).

5. Outline of statistical analysis

Analyses will be completed using Stata 18MP.

a. Descriptive analysis

Summary statistics of baseline characteristics of women and children will be presented with mean (SD) used to describe normally distributed continuous variables, median (IQR) for skewed continuous variables, and n (%) for categorical variables.

Population characteristics will be described for:

i) In-utero metformin exposure
ii) No in-utero metformin exposure

Table 1: Demographic and clinical characteristics by metformin exposure

Variable	No metformin in pregnancy<br>N=	Metformin in pregnancy<br>N=
**Maternal clinical and demographic characteristics**		
**Maternal age at birth,** Mean (SD)<br>Missing		
**Body mass index** (kg/m^2), Median (IQR)<br>Missing		
**Parity,** n (%)<br>- Nulliparous<br>- Multiparous<br>- Missing		
**Born outside Australia (yes),** n(%)		
**SEIFA quintile,** n (%)<br>1 – Most deprived<br>2<br>3<br>4<br>5 – Least deprived<br>Missing		
**Medical comorbidities,** n (%)<br>- Polycystic ovary syndrome<br>- Pre-eclampsia<br>- Gestational diabetes<br>- Type 1 diabetes<br>- Type 2 diabetes		
**Assisted reproductive technology (yes)**, n (%)<br>Missing		
**Child clinical and demographic characteristics**		
**Age at testing**, Mean (SD)<br>Missing, n (%)		
**Sex at birth,** n (%) female<br>Missing		
**Gestational age at birth (weeks),** Median (IQR)<br>Missing		
**Preterm birth (yes),** n (%)<br>Missing		
**Birthweight** (g), Mean (SD)<br>Missing, n (%)		
**Birthweight >97th centile,** n(%)		

© 2026 Gordon H et al. *JAMA Network Open*.

**Birthweight $<10^{th}$ centile,** n (%)		
**Mode of birth**, n (%)<br>- Unassisted vaginal birth<br>- Assisted vaginal birth<br>- Emergency caesarean section<br>- Elective caesarean section<br>- Missing		
**Highest parental education level,** n (%)<br>- Year 10 or below<br>- High school<br>- Trade/TAFE/Diploma/ Certificate<br>- Undergraduate university degree or above<br>- Missing		
**Language background other than English,** n, (%)<br>Missing		

Timing of first metformin exposure will be descriptively included.

b. Outcome analysis

Primary outcome: Overall developmental vulnerability in the AEDC (scoring $<10^{th}$ centile in ≥ 2 domains) by metformin exposure (exposed vs unexposed). This will be summarised using unadjusted and adjusted effect estimates and corresponding 95% confidence intervals (relative risk; risk difference) as outlined in Table 2.

Secondary outcome: Offspring developmental vulnerability (scoring $<10^{th}$ centile) in each of the five assessed developmental domains in the AEDC by metformin exposure (exposed vs unexposed).

Sensitivity analyses will assess:

1) The risk of overall developmental vulnerability based on timing of metformin exposure (first, second, third trimester) compared with no metformin use;
2) The risk of overall developmental vulnerability based on consistent metformin use, as defined by filling more than one metformin scripts during pregnancy, compared with no metformin use; and,
3) The risk of overall developmental vulnerability among metformin-exposed infants compared with no metformin use, with a cohort restricted to include only women with diabetes in pregnancy (gestational diabetes or type 2 diabetes). The most common indication for metformin in pregnancy is for management of diabetes.[15] In light of the relationship between diabetes and other factors that may influence childhood developmental outcomes such as high body mass index, advanced maternal age and ethnicity,[16] we will perform the analysis in a diabetic-only population to eliminate diabetes as a confounder.

All sensitivity analyses will be adjusted for relevant covariates, as described below.

Table 2: AEDC scores by metformin exposure

		**Metformin unexposed**	**Metformin exposed**	**Effect estimate (95% CI)**			
				**Unadjusted**		**Adjusted**	
**Developmental vulnerability** (scoring $<10^{th}$ centile in ≥ 2 domains), n (%)				RR	Percentage risk difference	RR	Percentage risk difference
**Domain-specific developmental vulnerability,** n (%)	Physical health and wellbeing						
	Social competence						
	Emotional maturity						
	Language and cognitive skills (school-based)						
	Communication skills and general knowledge						

Abbreviations: CI = Confidence interval; RR = Relative risk

© 2026 Gordon H et al. *JAMA Network Open*.

Covariates to be considered for inclusion in the both the propensity score selection and regression adjustment outcome models will be identified by the directed acyclic graphs (using DAGitty v3.1) and informed by the expert authorship team (Figures 1,2).

Adjustment models: Potential estimators are (i) selection model, using inverse probability weighting (IPW); (ii) outcome model, using regression adjustment; (iii) doubly robust inverse probability weighted regression adjustment (IPWRA) models, (iv) Propensity score stratification with stratum effects pooled to estimate an overall treatment effect. Under correct specification, all the estimators should produce similar results. We will focus on IPWRA; the selection model will be included in the IPW and outcome in regression (RA) model specification.

We will also perform regression adjustment, inverse probability weight and multivariate logistic regression, and propensity score stratification with regression adjustment separately to examine the robustness of our effect estimate using different approaches to analysis. We may be unable to perform propensity score stratification eg. due to an unequal distribution of propensity scores resulting in empty strata when coarsened to a minimum of five strata.[17] In this instance, we will perform propensity score matching using nearest neighbour matching at a ratio of one case to five controls to examine the robustness of our effect estimate.

c. Approach to missing data

Detailed description of missing data will be provided. This will include the proportion of missingness for each covariate and the five outcome domains of each assessment domain of the AEDC and the total number of observations with complete data. Presentation of missing data patterns will use graphical means of summarization. This will include assessment of exposure distributions for (i) assessment AEDC exempt (missing not at random)/non exempt children and (ii) special needs (missing not at random)/no special needs. If distributions are close then consideration to exclude these children may be made – as unable to effect estimates.

We don't anticipate missing exposure data.

We will consider that missing AEDC results from the point of view of both missing not at random and missing at random mechanisms. For our primary analysis we will treat this outcome missingness conservatively by deterministically imputing all missing AEDC scores as 'vulnerable'; a sensitivity analyses will be undertaken excluding any child with any missing outcomes. If the distribution of missingness is found to be non-differential between exposure (*vide supra*) then these observations can be excluded from the analysis.

Covariates that are included in the imputation models are: the five AEDC domain outcomes, all covariates included in selection and outcome models irrespective of missingness, exposure and auxiliary covariates associated with the outcome.

Multiple imputation will be performed using fully conditional specification and the imputed datasets will then be used for the outcome analysis. It is important that the models used in the imputation phase are compatible with those used for ATE estimation. This requirement means that choice of imputation models requires knowledge of the analysis model and the covariates used in both selection and/or outcome models to ensure that there is compatibility between the two. This is an open area of active research with methods chosen based upon current recommendations in the literature.

Standard imputation diagnostics will then be performed to assess the quality of the imputed datasets.

d. **The Bartlett and von Hippel method (*vide infra*) will be used to provided population effect standard errors.**[18] Using this technique the number of bootstrapped samples will be set at 500, the FCS imputations set at 2 with a burn-in of 10.

e. **Selection model using Inverse Probability Weights (IPW).**

- <u>We will model exposure assignment using inverse probability weighted propensity scores derived from a logistic exposure model.</u>
- Adequacy of balance achieved by PS weighting will be assessed using: (i) standardized mean difference for model covariates and variance estimates between exposure groups and (ii) propensity scores overlap, to assess the positivity assumption.
- A finding of lack of positivity will lead to measures such as: addition of interaction terms; different exposure models; use of alternate weighting structures (overlap or stabilized); and lastly propensity score-based trimming with the aim of achieving overlap if the propensity score distributions.

© 2026 Gordon H et al. *JAMA Network Open*.

Outcome model using Regression Adjustment (RA)

- In addition to the selection model, we will use a regression adjustment outcome model.
- Outcome modifiers such as age of testing, year of test and sex at birth will be included in this model to account for covariate – outcome associations.

Inverse Probability Weighted (IPW) Regression Adjustment (RA) model (IPWRA)

The doubly robust method combines the IPW selection model with RA outcome model. This provides two opportunities for correct model specification and may provide valid estimates if only one of the submodels are mis-specified.

Population effect estimate using the von Hippel method:

If the imputation and analysis models are compatible and correctly specified then synthesis using Rubin's rules provides valid variance estimates and confidence intervals with nominal coverage. However, when the imputation and analysis procedures are uncongenial and/or misspecified, this is not correct.

And

Probability weighting has the potential to induce within-subject correlation in outcome in the weighted sample resulting in biased low SE. While a robust SE setting in the analysis model does account for this lack of independence it doesn't account for the fact that the propensity score is estimated rather than know with absolute certainty.

Alternatives that account for these two issues include: bootstrapping, where the propensity score is estimated within each bootstrap sample and thus the bootstrap estimate of the standard error incorporates sampling variability in the estimated propensity score (BootImpute)[19] and BootImpute von Hippel[18] where the ATE variance estimate is calculated from a ANOVA model and requires a smaller number of bootstrap samples and imputation.

We will integrate IPW with multiple imputation to calculate the pooled effect will be performed using the BootMI von Hippel method.

In brief we obtain B (B=500) bootstrapped samples from the observational data; for each of the B bootstrapped samples impute M datasets (M = 2); the effect point estimate is calculated as the mean over all B times M complete datasets; a one way ANOVA models is used estimate the two variance components (within and between bootstrap samples) which are then used to estimate the standard error and construct associated 95% confidence intervals incorporating Satterthwaite's degrees of freedom correction.

© 2026 Gordon H et al. *JAMA Network Open*.

eFigure 1. Directed Acyclic Graph: Metformin in Pregnancy and Offspring Development

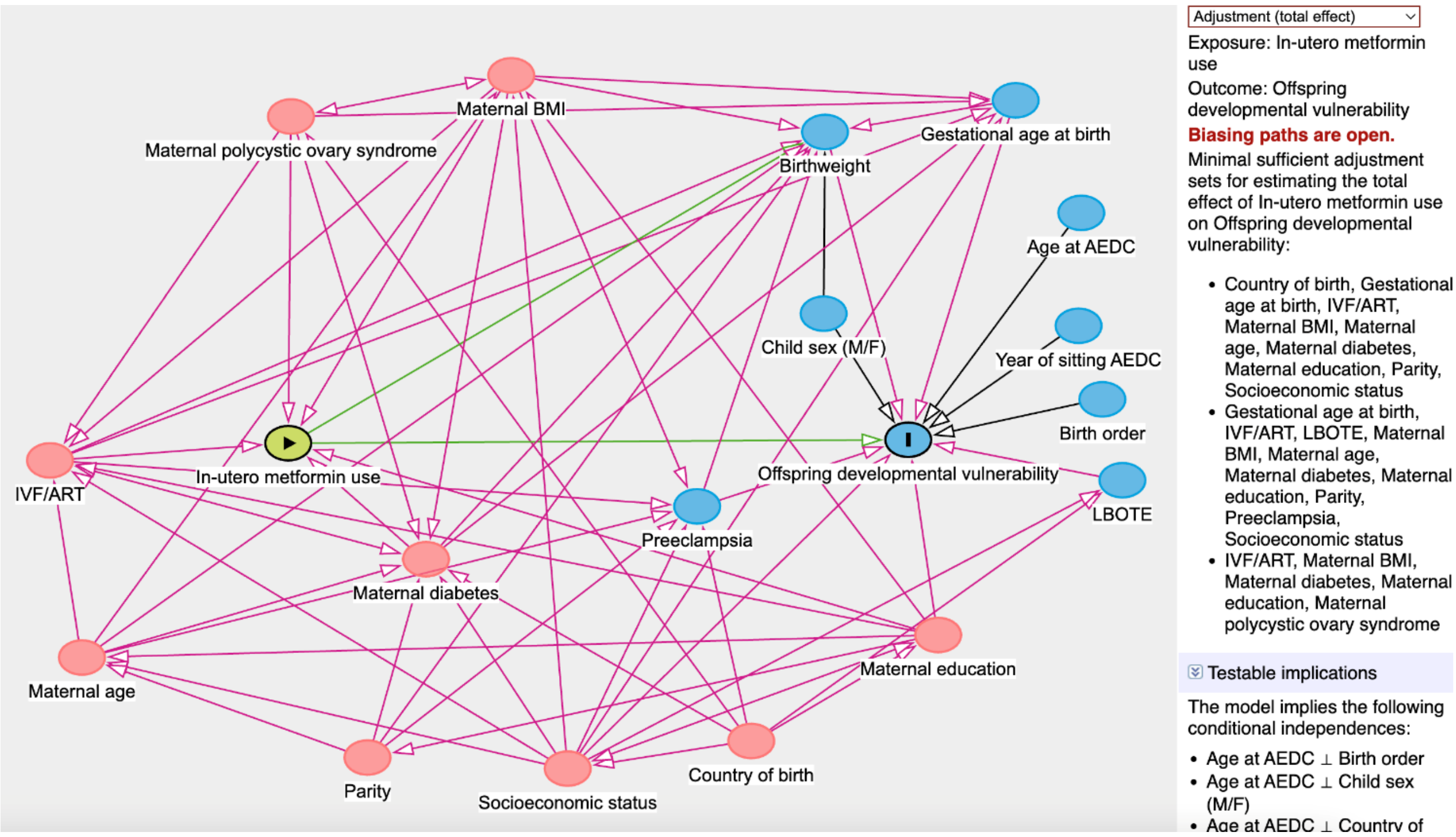

eFigure 2. Directed Acyclic Graph: Sensitivity Analysis—Metformin in Pregnancy Among Women With Diabetes (Type 2, Gestational Diabetes) and Offspring Development

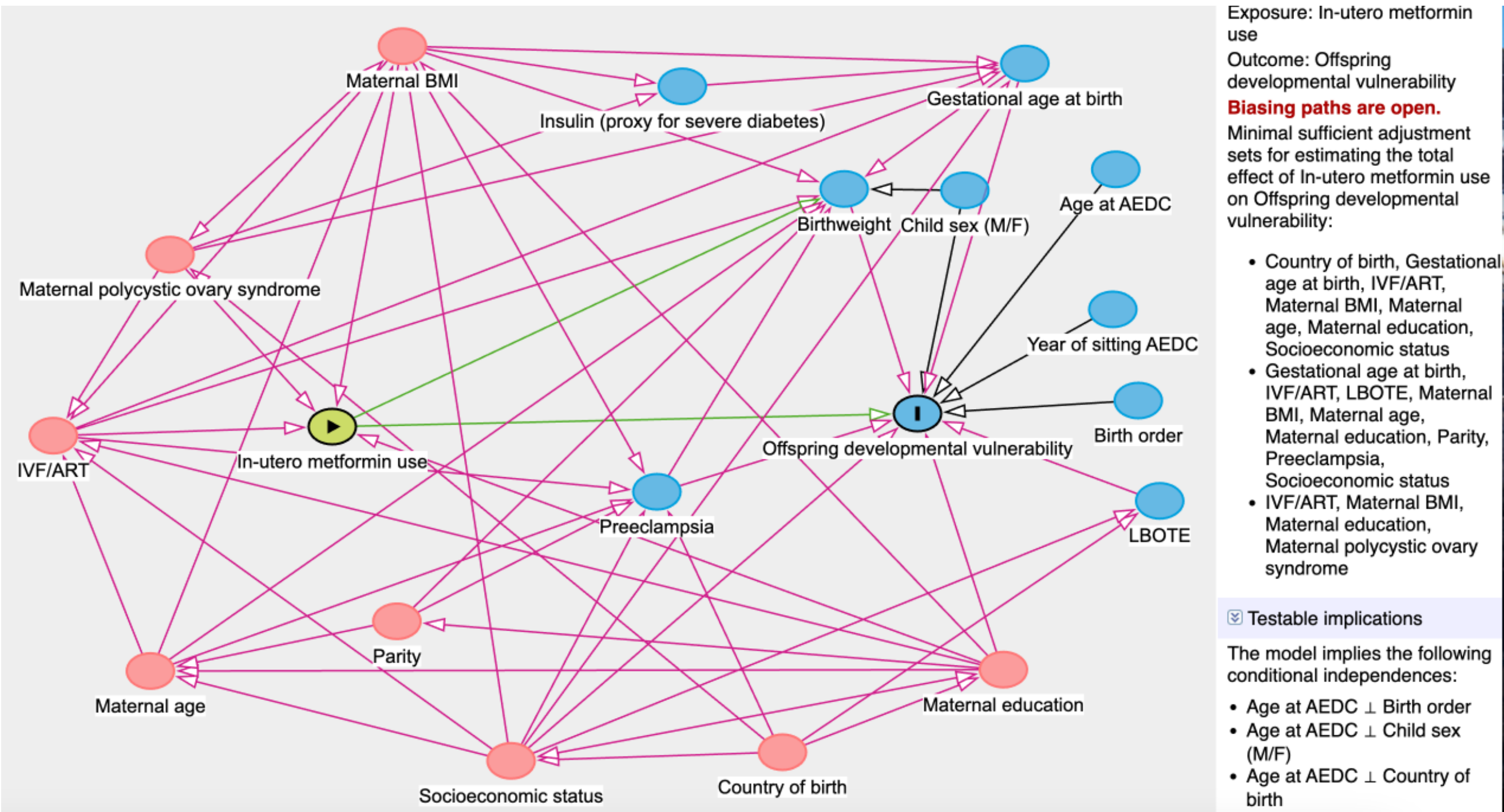

© 2026 Gordon H et al. *JAMA Network Open*.

eReferences

1. The Royal Australian College of General Practitioners. *Management of Type 2 Diabetes: A Handbook for General Practice*. RACGP; 2020.
2. Charles B, Norris R, Xiao X, Hague W. Population Pharmacokinetics of Metformin in Late Pregnancy. *Ther Drug Monit*. 2006;28(1):67-72. doi:10.1097/01.ftd.0000184161.52573.0e
3. Goh JEL, Sadler L, Rowan J. Metformin for gestational diabetes in routine clinical practice. *Diabet Med*. 2011;28(9):1082-1087. doi:10.1111/j.1464-5491.2011.03361.x
4. Tarry-Adkins JL, Ozanne SE, Aiken CE. Impact of metformin treatment during pregnancy on maternal outcomes: a systematic review/meta-analysis. *Sci Rep*. 2021;11(1):9240. doi:10.1038/s41598-021-88650-5
5. Charlton RA, Klungsøyr K, Neville AJ, et al. Prescribing of Antidiabetic Medicines before, during and after Pregnancy: A Study in Seven European Regions. Staiger H, ed. *PLOS ONE*. 2016;11(5):e0155737. doi:10.1371/journal.pone.0155737
6. Australian Government Department of Health TGA. Prescribing medicines in pregnancy database. https://www.tga.gov.au/prescribing-medicines-pregnancy-database
7. Gordon HG, Atkinson JA, Tong S, et al. Metformin in pregnancy and childhood neurodevelopmental outcomes: a systematic review and meta-analysis. *Am J Obstet Gynecol*. Published online March 2024:S0002937824004307. doi:10.1016/j.ajog.2024.02.316
8. Rowan JA, Rush EC, Plank LD, et al. Metformin in gestational diabetes: the offspring follow-up (MiG TOFU): body composition and metabolic outcomes at 7–9 years of age. *BMJ Open Diabetes Res Care*. 2018;6(1):e000456. doi:10.1136/bmjdrc-2017-000456
9. Oner M, Chen MC, Cheng PT, et al. Impact of metformin on neocortical development during pregnancy: Involvement of ERK and p35/CDK5 pathways. *Chemosphere*. 2024;358:142124. doi:10.1016/j.chemosphere.2024.142124
10. Van Dam JM, Garrett AJ, Schneider LA, et al. Reduced Cortical Excitability, Neuroplasticity, and Salivary Cortisol in 11–13-Year-Old Children Born to Women with Gestational Diabetes Mellitus. *EBioMedicine*. 2018;31:143-149. doi:10.1016/j.ebiom.2018.04.011
11. Flood MM, McDonald SJ, Pollock WE, Davey MA. Data accuracy in the Victorian Perinatal Data Collection: Results of a validation study of 2011 data. *Health Inf Manag J*. 2017;46(3):113-126. doi:10.1177/1833358316689688
12. Australian Institute of Health and Welfare (AIHW). *Diabetes: Australian Facts*.; 2023. https://www.aihw.gov.au/getmedia/b1d366a5-edef-4356-98df-2ba74d5cb60c/diabetes-australian-facts.pdf?v=20240206071723&inline=true
13. Australian Institute of Health and Welfare. *Diabetes Prevalence in Australia: An Assessment of National Data Sources*.; 2009:11.
14. The Australian Early Development Census. ABOUT THE AEDC. Published online 2022. Accessed January 28, 2025. https://www.aedc.gov.au/about-the-aedc
15. Lindsay RS, Loeken MR. Metformin use in pregnancy: promises and uncertainties. *Diabetologia*. 2017;60(9):1612-1619. doi:10.1007/s00125-017-4351-y
16. Zhang C, Rawal S, Chong YS. Risk factors for gestational diabetes: is prevention possible? *Diabetologia*. 2016;59(7):1385-1390. doi:10.1007/s00125-016-3979-3
17. Benedetto U, Head SJ, Angelini GD, Blackstone EH. Statistical primer: propensity score matching and its alternatives†. *Eur J Cardiothorac Surg*. 2018;53(6):1112-1117. doi:10.1093/ejcts/ezy167
18. Hippel PT von, Bartlett JW. Maximum Likelihood Multiple Imputation: Faster Imputations and Consistent Standard Errors Without Posterior Draws. *Stat Sci*. 2021;36(3):400-420. doi:10.1214/20-STS793
19. Bartlett JW, Hughes RA. Bootstrap inference for multiple imputation under uncongeniality and misspecification. *Stat Methods Med Res*. 2020;29(12):3533-3546. doi:10.1177/0962280220932189

© 2026 Gordon H et al. *JAMA Network Open*.
