# Supplementary material for: Metformin Exposure in Pregnancy and Childhood Developmental Outcomes
Source: JAMA Netw Open. 2026 Sep 30;9(9):e2637054. doi: 10.1001/jamanetworkopen.2026.37054 (PMC13628313; doi:10.1001/jamanetworkopen.2026.37054)
Supplement: Supplementary file 2 [file jamanetwopen-e2637054-s002.pdf]

Data Sharing Statement

Data
Data available: No

Additional Information
Explanation for why data not available: The data underlying this study are not publicly available. Access is restricted due to ethical and governance requirements and subject to approval by the relevant Human Research Ethics Committees and data custodians.
